# Novel Therapeutic Targets in Liver Fibrosis

**DOI:** 10.3389/fmolb.2021.766855

**Published:** 2021-11-05

**Authors:** Jinhang Zhang, Qinhui Liu, Jinhan He, Yanping Li

**Affiliations:** ^1^ Laboratory of Clinical Pharmacy and Adverse Drug Reaction, National Clinical Research Center for Geriatrics, West China Hospital, Sichuan University, Sichuan, China; ^2^ Department of Pharmacy, State Key Laboratory of Biotherapy, West China Hospital, Sichuan University, Sichuan, China

**Keywords:** liver fibrosis, hepatic stellate cells, therapeutic targets, molecular mechanism, non-alcocholic fatty liver disease

## Abstract

Liver fibrosis is end-stage liver disease that can be rescued. If irritation continues due to viral infection, schistosomiasis and alcoholism, liver fibrosis can progress to liver cirrhosis and even cancer. The US Food and Drug Administration has not approved any drugs that act directly against liver fibrosis. The only treatments currently available are drugs that eliminate pathogenic factors, which show poor efficacy; and liver transplantation, which is expensive. This highlights the importance of clarifying the mechanism of liver fibrosis and searching for new treatments against it. This review summarizes how parenchymal, nonparenchymal cells, inflammatory cells and various processes (liver fibrosis, hepatic stellate cell activation, cell death and proliferation, deposition of extracellular matrix, cell metabolism, inflammation and epigenetics) contribute to liver fibrosis. We highlight discoveries of novel therapeutic targets, which may provide new insights into potential treatments for liver fibrosis.

## Introduction

### Liver Fibrosis

Liver fibrosis is a repair response to chronic liver injury caused by various pathogenic factors, and it is characterized mainly by the excessive accumulation of extracellular matrix (ECM), especially collagen fibers (Reeves and Friedman, 2002). If the pathogenic factor is not removed, liver fibrosis can progress to liver cirrhosis and even hepatocellular carcinoma, which elevates risk of mortality.

Liver fibrosis has become one of the most common liver diseases worldwide, and it has also become one of the leading indications for liver transplantation. The global prevalence of nonalcoholic fatty liver disease (NAFLD) is 25.24%, and its prevalence is particularly high in the Middle East, South America and Asia. Just over half of patients (59.1%) with NAFLD, and in particular 40.76% of patients with liver fibrosis, progress to nonalcoholic steatohepatitis (NASH) (Younossi et al., 2016). Liver disease accounts for approximately 2 million deaths per year worldwide, among which 1 million deaths occur due to complications from cirrhosis, which is currently the 11th most common cause of death globally (Asrani et al., 2019).

Alcohol abuse, chronic viral hepatitis, obesity, autoimmune hepatitis, metabolic syndrome and cholestasis are the most common causes of liver fibrosis (Bataller and Brenner, 2005). If the pathogenic factors are acute or self-limiting, wound-healing responses are transient, and the liver architecture can return to normal. When the factors persist, the inflammatory phase begins and hepatic stellate cells (HSCs) activate, leading to ECM deposition and destruction of the liver architecture. The pathogenesis of liver fibrosis is complicated and involves multiple types of liver cells and inflammatory reactions. Its main pathological features are collagen deposition and damage of liver structure. Numerous studies of anti-fibrosis targets have focused mainly on collagen deposition and various types of hepatic cells.

## Hepatic Cells in Liver Fibrosis

**FIGURE 1:**
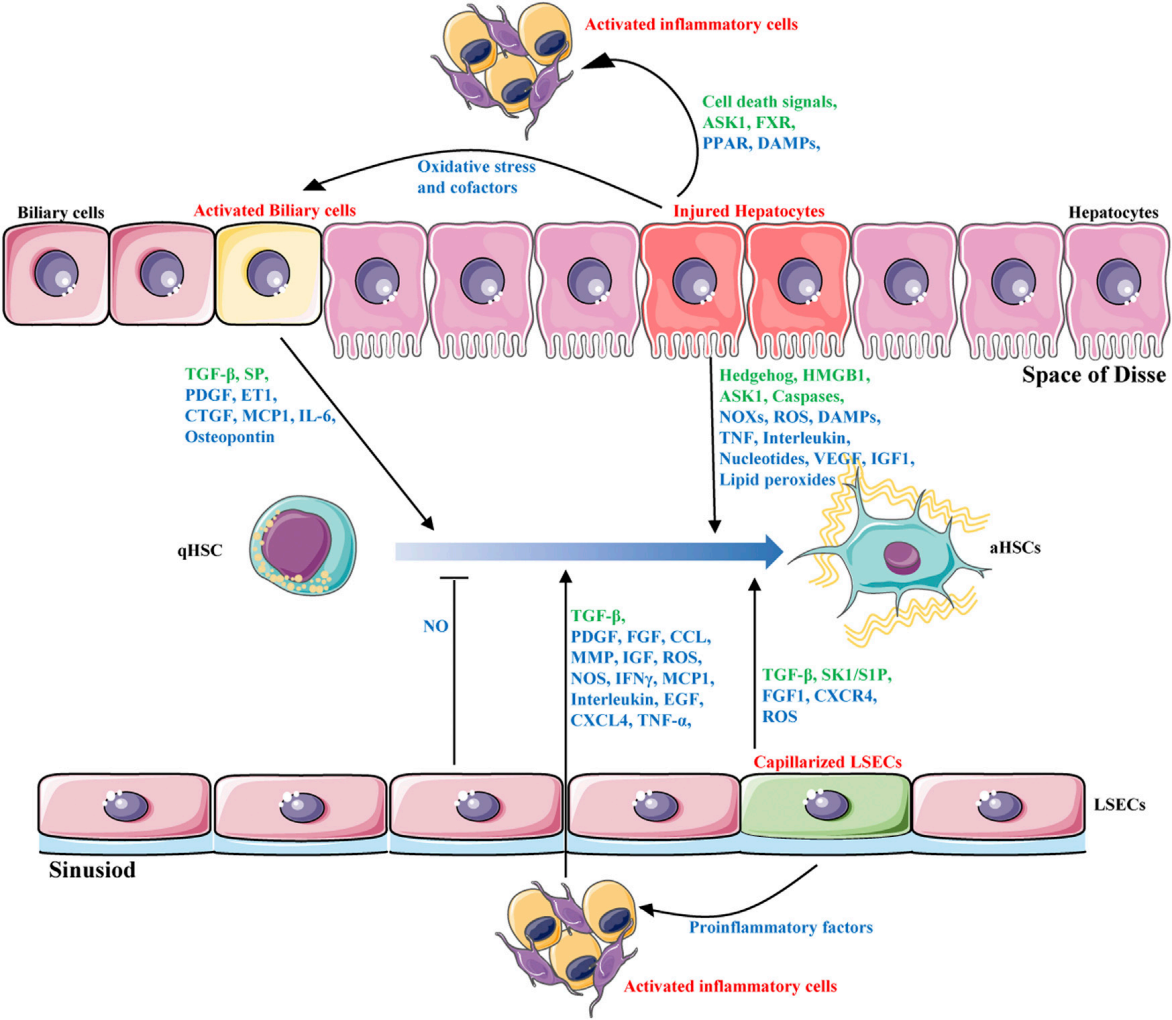
Intercellular crosstalk during liver fibrosis. HSCs activation is the major driver of liver fibrosis that depends on the interaction with other hepatic cells, including hepatocytes, biliary cells, liver sinusoidal endothelial cells and inflammatory cells. These cells interact with each other and promote or inhibit the activation of HSCs through production of hormones, cytokines(blue) and other signalling molecules(green).

Liver fibrosis is a complex process of liver self-repair that involves multiple types of hepatic cells. Intercellular crosstalk within the liver microenvironment is critical for the maintenance of normal hepatic functions and cell survival (Marrone et al., 2016). The chronic presence of external pathological factors can injury hepatocytes; activate inflammatory cells such as macrophages; promote infiltration of lymphocytes; trigger proliferation of sinusoidal endothelial cells and capillarization of sinusoidal endothelial cells, blocking perfusion between blood and liver cells and causing abdominal aortic hypertension; and activate bile duct cells (Boyer-Diaz et al., 2021). These changes ultimately lead to the activation of HSCs, the most important source of myofibroblasts, to synthesize excess ECM, resulting in liver fibrosis. HSC activation is considered central in liver fibrosis. However, other types of liver cells also play important roles in fibrosis. Indeed, HSCs activation depends on the interaction with other hepatic cells, including hepatocytes, liver sinusoidal endothelial cells, inflammatory cells and biliary cells (Figure 1). These cells interact with each other and promote or inhibit the activation of HSC through production of cytokines and other signalling molecules. Targeting the crosstalk between HSCs and other hepatic cells might be a novel option for liver fibrosis treatment.

### Hepatic Stellate Cells

Activation of HSCs, often referred to as their “*trans*-differentiation”, is the major cellular source of matrix protein-secreting myofibroblasts, which are the major driver of liver fibrogenesis (Higashi et al., 2017; Tsuchida and Friedman, 2017; Cai et al., 2020). HSCs, which are also called vitamin A-storing cells, lipocytes, interstitial cells, fat-storing cells or Ito cells, exist in the space between parenchymal cells and liver sinusoidal endothelial cells of the hepatic lobule. They store 50–80% of the total vitamin A in the body; they store the vitamin A in the form of retinyl palmitate in lipid droplets in the cytoplasm (Senoo et al., 2010). HSCs in the space of Disse are also thought to contribute reversibly to portal hypertension (McConnell and Iwakiri, 2018).

Stimulating HSCs with external factors such as lipid peroxides or pro-fibrotic cytokines leads them to lose lipid droplets, proliferate, and transform into myofibroblasts. The cells then begin to produce ECM and they acquire contractile, pro-inflammatory, and fibrogenic properties (Bataller and Brenner, 2005). The disordered accumulation of ECM results in scar and liver fibrosis (Gandhi, 2017). Many current anti-fibrosis treatments aim to prevent HSCs from contributing to fibrosis, such as by blocking their activation by external factors, inhibiting their proliferation, promoting their apoptosis (Trivedi et al., 2021), and preventing their adoption of a high metabolic state (Du et al., 2018; Khomich et al., 2019).

**FIGURE 2:**
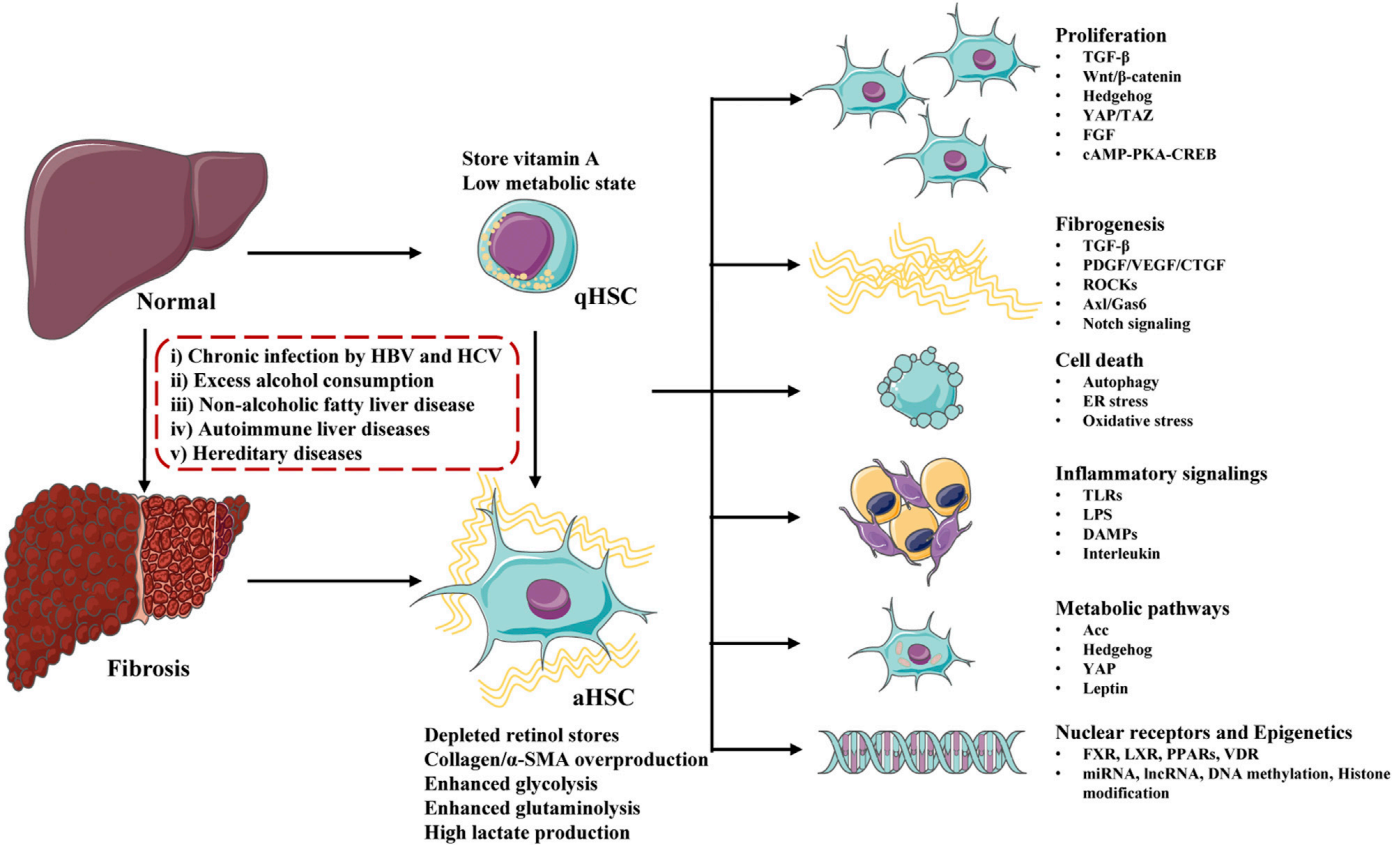
Major molecules and signaling pathways regulating hepatic stellate cells (HSCs) activation and liver fibrosis. Undergo physiological conditions, HSCs exist as a quiescent phenotype (qHSCs), which store vitamin A and stay in a low metabolic state. After stimulating by a serious of factors (including chronic infection, excess alcohol consumption, non-alcoholic fatty liver disease, autoimmune liver diseases and hereditary diseases), qHSCs activate into activated HSCs (aHSCs) and liver fibrosis develop. Activated HSCs lose stored retinol, produce excessive collagen/α-SMA and stay at a high metabolic state. HSCs activation is regulated by a number of signaling pathways and molecules, including proliferation, fibrogenesis, cell death, inflammatory signalings, metabolic pathways, nuclear receptors and epigenetics.

The major signaling pathways involved in HSCs activation contains: growth factors and ligand-receptor signaling pathways (Wnt/β-catenin, Hedgehog, YAP/TAZ, FGF, cAMP-PKA-CREB), profibrogenic response pathways (TGF-β, PDGF/VEGF/CTGF, ROCKs, Axl/Gas6, Notch, renin angiotensin system), cell death signaling (autophagy, ER stress, oxidative stress), immune-related signaling (TLRs, LPS, DAMPs, interleukin), metabolic regulated pathways (Acc, Hedgehog, YAP, leptin), Nuclear receptors (FXR, LXR, PPARs, VDR) and epigenetic changes (miRNA, lncRNA, DNA methylation, histone modification) (Figure 2). Although some clinical drugs were found to be positive for liver fibrosis treatment in clinical trails including PPAR-γ agonist (pioglitazone), angiotensin receptor blockers (losartan, telmisartan, olmesartan and candesartan), and glucagon-like peptide-1 receptor agonists (liraglutide), the safety and effect of these drugs need to be further confirmed (Georgescu, 2008; Mantovani et al., 2021). Clinical existing drugs like angiotensin receptor blockers, PPAR-γ agonist may be accompanied by side effects due to their wide range of effects, and they may not be suitable for the treatment of simple liver fibrosis. It is necessary to find new therapeutic targets.

Myofibroblasts, which are not present in the healthy liver, are activated in response to liver injury (Friedman, 2008; Kisseleva and Brenner, 2021). They form from resident mesenchymal cells, epithelial cells (e.g. hepatocytes and cholangiocytes), endothelial cells, bone marrow stem cells, portal fibroblasts and HSCs (Wynn, 2008). The most important characteristic of liver fibrosis is the excessive deposition of ECM, in which myofibroblasts play the most important role (Karsdal et al., 2016). This makes ECM production a primary target for anti-fibrotic therapy. Inflammation often accompanied liver injury (Koyama and Brenner, 2017), which involves the production and release of such cytokines as CTGF, PDGF, and TGF-β. These cytokines activate myofibroblasts to produce abundant ECM (Wynn, 2008), inducing liver fibrosis.

### Hepatocytes

Hepatocytes are the most important parenchymal cells in liver; they account for more than 80% of all liver cells. Together with cholangiocytes, hepatocytes help maintain liver homeostasis (Dai et al., 2020). Damage to hepatocytes, together with subsequent inflammatory and fibrogenic signaling cascades, is thought to trigger fibrosis (Tu et al., 2017). After damage by microenvironmental factors, hepatocytes secrete pro-inflammatory and pro-fibrotic factors, activating inflammatory cells and HSCs, in turn promoting fibrosis (Wang et al., 2016a; Zhang et al., 2019; An et al., 2020). The epithelial‐mesenchymal transition (EMT) in hepatocytes promotes the progression of liver fibrosis. Fructose induces hepatocytes to upregulate fibroblast‐specific protein1 and vimentin, while downregulating E‐cadherin, thereby promoting EMT (Cicchini et al., 2015; Song et al., 2019).

In early-stage liver disease, if liver cell damage can be reversed, then hepatocytes can be promoted and liver fibrosis reversed. This is the aim of hepatoprotective drugs currently used in the treatment of liver diseases. In late-stage liver disease, in contrast, it is often impossible to inhibit the death of hepatocytes. This may be because HSCs are in a highly activated state, and they can secrete pro-fibrotic factors. Drugs that fail to target liver cells selectively and that instead are taken up by HSCs can promote HSC proliferation and inhibit their apoptosis. The problem of this “two-way” action must be taken into account when designing treatments that promote apoptosis or inhibit proliferation.

### Liver Endothelial Cells

LSECs act as permeable barriers and portal pressure regulators, they mediate the transcript of nutrients, they recruit lymphocytes from the blood, and they secrete cytokines and growth factors from their sinusoidal side (Asahara et al., 1999). LSECs have the highest endocytotic capacity of all human cells (Poisson et al., 2017). They also interact with HSCs and hepatocytes, and they are critical to maintain HSC quiescence and regenerate hepatocytes (Hu et al., 2014), thus inhibiting intrahepatic vasoconstriction and fibrosis. LSECs maintain HSC quiescence via a pathway that is stimulated by vascular endothelial growth factor (VEGF) and that depends on nitrous oxide (NO) (Asahara et al., 1999; Marrone et al., 2013; DeLeve, 2015). In chronic liver injury, LSECs undergo capillarization, they downregulate eNOS and NO synthesis, and they secrete profibrogenic and proinflammatory cytokines such as TGF-β1, PDGF, TNF-α and IL-6, thereby promoting liver fibrosis (Lafoz et al., 2020).

### Cholangiocytes

Cholangiocytes are epithelial cells lining the intra- and extra-hepatic bile ducts; they are heterogeneous in size and function and mediate solute transport processes that determine the composition and flow of bile (Banales et al., 2019). Their dysfunction lies at the heart of cholangiopathies. During biliary disease, various pathological stimuli such as gastrointestinal hormones, bile acids, angiogenic factors, and nerve growth factor can activate cholangiocytes, leading to biliary proliferation, known as a ductular reaction. The result is an epigenetically-regulated transcriptional program involving secretion of TGF-β1, CTGF, p16, CCL2 and SA-β-gal, ultimately leading to a profibrogenic micro-environment, HSC activation, and enhanced liver fibrosis (Zhou et al., 2018; Elssner et al., 2019; Jalan-Sakrikar et al., 2019). The ductular reaction contributes to the initiation and progression of liver fibrosis (Glaser et al., 2009).

Many recent studies have explored the role of cholangiocytes in liver fibrosis. For example, non-canonical NF-κB can contribute to cholangiocyte proliferation and the ductular reaction, accelerating liver fibrosis (Elssner et al., 2019). The long non-coding RNA H19, present in exosomes from cholangiocytes, can activate HSCs and promote cholestatic liver fibrosis (Liu et al., 2019a). Knockout of the secretin receptor reduces biliary damage and liver fibrosis by slowing cholangiocyte senescence (Zhou et al., 2018). These findings suggest that targeting the activation of cholangiocytes and the ductular reaction can mitigate biliary fibrosis.

### Inflammatory Cells

The acute inflammation that arises in response to liver injury is thought to help mitigate infection and promote liver repair and regeneration (Karin and Clevers, 2016). Chronic inflammation, in contrast, is detrimental and contributes to liver fibrosis through the involvement of multiple types of inflammatory cells, including Kupffer cells, recruited macrophages, neutrophils, Th17 cells and Tregs (Berumen et al., 2021; Wen et al., 2021). Macrophages play a dual role in the progress of fibrosis. M1 macrophages produce inflammatory cytokines, while M2 macrophages regulate inflammatory responses and secret matrix metalloproteases (MMPs), the main enzymes that degrade ECM, thereby reversing fibrosis (Pradere et al., 2013; Luo et al., 2019). Thus, the balance between M1 and M2 macrophages influences whether fibrosis progresses or not (Sica et al., 2014). Interestingly, recent studies have suggested that M1, but not M2 macrophages, inhibit liver fibrogenesis by recruiting endogenous macrophages and “polarizing” them into a restorative Ly6C^lo^ phenotype, which secrets high levels of MMPs for collagen degradation, as well as high levels of hepatocyte growth factor for hepatocyte proliferation (Ramachandran et al., 2012; Ma et al., 2017).

Kupffer cells, the resident macrophages of the liver, play a central role in liver inflammation. They are resident macrophages that localize within the lumen of the liver sinusoids, and they account for about 30% of sinusoidal cells (Bouwens et al., 1986; Koyama and Brenner, 2017). In response to hepatocyte injury, Kupffer cells become active and secret pro-inflammatory and pro-fibrosis factors. TGF-β, which is secreted mainly by Kupffer cells and plays a key role in liver fibrosis (Xu et al., 2020), binds to a receptor on HSCs to activate them and induce production of collagen (Wang et al., 2019a). Hepatic macrophages also enhance liver fibrosis through the release of IL-1β, TNF-α, CCL2 and PDGF. During liver steatosis, neutrophils and Kupffer cells release reactive oxygen species (ROS), promoting HSC activation and liver fibrosis (Jiang et al., 2012; Dat et al., 2021). Th17 cells produce IL-17, which activates Kupffer cells and express the proinflammatory cytokines IL-6, IL-1β and TNF-α. IL-17 also directly activates HSCs and promotes collagen production via the STAT3 pathway (Meng et al., 2012). Th22 cells, for their part, produce IL-22, which drives TGF-β-dependent liver fibrosis (Fabre et al., 2018).

In this way, many types of inflammatory cells and complex molecular pathways are involved in liver fibrosis. Future studies aiming to treat liver fibrosis by targeting inflammatory cells should cautiously consider the potentially complex effects of such treatment.

## New Therapeutic Targets

Liver fibrosis can be reversed in early stages if the pathological insult can be removed. Here we summarize recent reports on signaling pathways that contribute to liver fibrosis and on efforts to target such pathways as a therapeutic strategy.

### TGF-β Signaling

**FIGURE 3:**
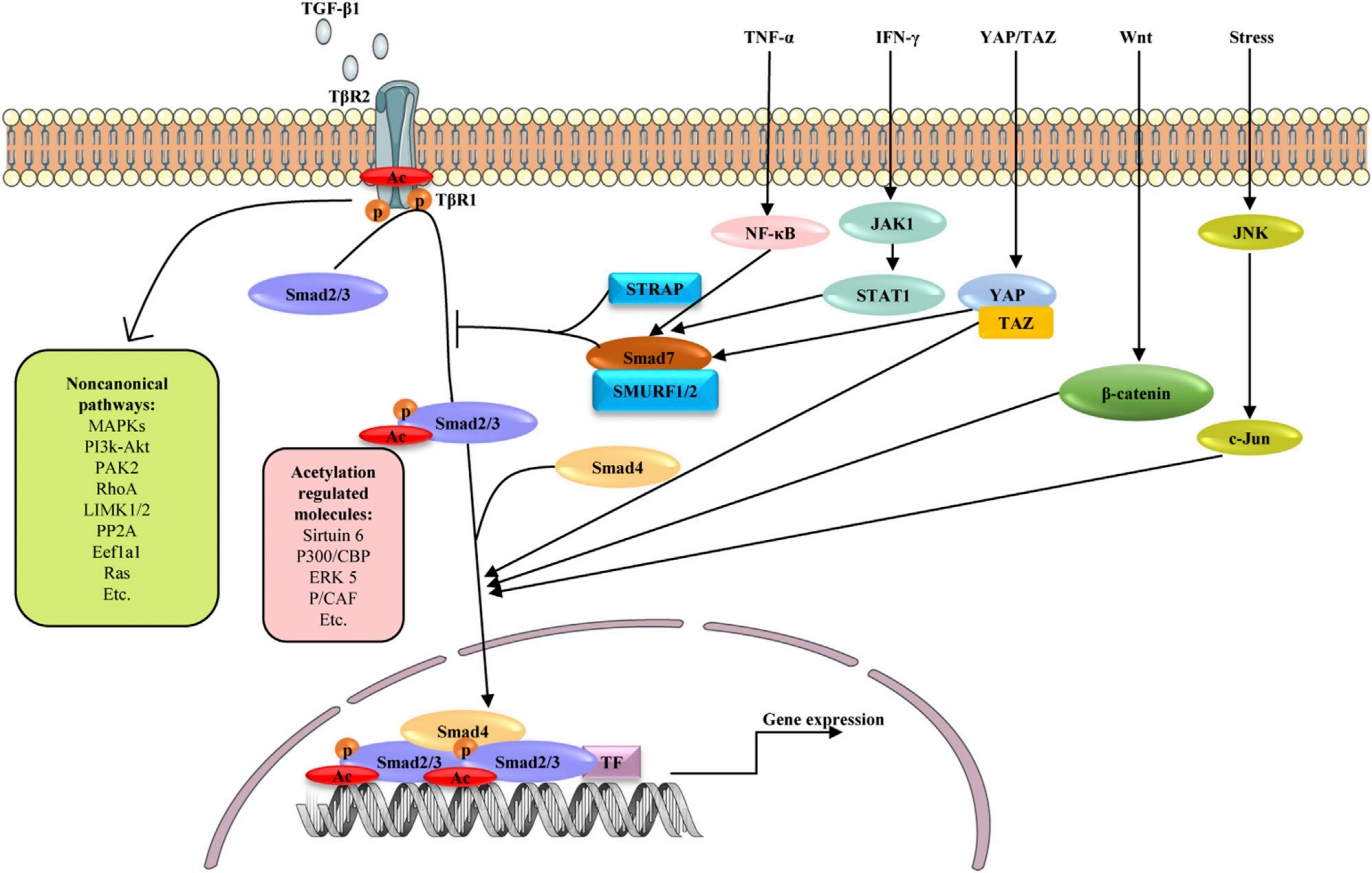
Schematic diagram depicting possible mechanisms involved in the fibrogenesis of TGF-β signaling and its crosstalk with other signaling pathways. Signaling starts with TGF-β binding to TGFβR2 (TβR2), which activates TGFβR1 (TβR1). The active TβR1 phosphorylates and acetylates Smad2/3, which complex with Smad4, translocate into nucleus and promote profibrotic genes expression. As another active form of Smad2/3, acetylated Smad2/3 are reported to be regulated by Sirtuin6, P300/CBP, ERK5 and P/CAF. Smad7 negatively regulates TGF-β signaling through competing with Smad2/3 for interaction with TβR1 in the presence of STRAP and SMURF1/2. TGF-β can also activate non-canonical TGF-β pathways, including MAPK, PI3K/AKT, PAK2, RhoA, LIMK1/2, *p*P2A, Eefla1and Ras pathways. In addition to canonical and non-canonical pathways, TGF-β/Smad signaling shares crosstalk with other signaling pathways in liver fibrosis, including TNF-α, IFN-γ, YAP/TAZ, Wnt and JNK signaling. These pathways influence TGF-β signaling by affecting the activation and nuclear translocation of Smad2/3. TF: transcription factors, p: phosphate group, Ac: acetylate group.

TGF-β signaling is a core regulator of fibrosis, and it can induce fibrosis via canonical and non-canonical (non-Smad) pathways (Figure 3) (Finnson et al., 2020). In both cases, myofibroblasts are activated, leading to excessive ECM production and inhibition of ECM degradation (Meng et al., 2016). TGF-β binds to its cognate receptor TGF-β type II receptor, inducing the nuclear translocation of Smad2 and Smad3, which regulate the transcription of fibrotic target genes (Zhang et al., 2021a).

In the canonical pathway, Smad2/3 is activated by phosphorylation but potentially also by lysine acetylation to promote liver fibrosis (Bugyei-Twum et al., 2018; Wang et al., 2019a; Zhong et al., 2020; Zhang et al., 2021a). The chromatin deacylase Sirtuin 6 is also an important regulator of liver fibrosis through its influence on metabolism, DNA repair, gene expression, and mitochondrial biology (Andrew et al., 2020). Sirtuin6 deficiency induces aging-dependent fibrosis in liver and other organs in mice. Sirtuin6 may deacetylate Smad3 as well as Lys-9 and Lys-56 in histone 3 to repress the expression of key TGF-β signaling genes (Maity et al., 2019). By deacetylating lysines 333 and 378 of Smad3, sirtuin6 may inhibit Smad3 activity, protecting against liver fibrosis (Zhong et al., 2020). Like Smad3, Smad2 is also a major acetylated substrate of sirtuin6. Sirtuin6 deacetylates lysine 54 on Smad2, reducing TGF-β/Smad2 signaling in HSCs and thereby alleviating liver fibrosis. By deacetylating Smad2, sirtuin6 influences its phosphorylation and nuclear translocation (Zhang et al., 2021a). These findings suggest that TGF-β signaling is a master regulator of fibrosis and warrants multilayer control, and that sirtuin6 may regulate TGF-β signaling at multiple levels.

TGF-β also regulates other signaling pathways through non-Smad signaling pathways, such as pathways involving Wnt/β-catenin, MAPK, mTOR, IKK, PI3K/Akt, and Rho GTPase, thereby contributing to liver fibrosis (Zhang, 2017; Mi et al., 2019). TGF-β-mediated upregulation of FoxO3a and the DNA demethylase TET3 in HSCs facilitates hepatic fibrogenesis (Xu et al., 2020; Kim et al., 2021). TGF-β can also regulate proteasomal degradation of EZH2 in cholangiocytes, supporting biliary fibrosis (Jalan-Sakrikar et al., 2019). TGF-β upregulates hyaluronan (HA) synthase 2, leading to increased production of HA, a major extracellular matrix glycosaminoglycan and biomarker for cirrhosis. HA promotes the fibrogenic, proliferative, and invasive properties of HSCs via pathways involving the receptors CD44, Toll-like receptor 4 (TLR4), and Notch1 (Yang et al., 2019). TGF-β1 activates the p65/MAT2A pathway to decrease levels of S-adenosylmethionine, thereby facilitating liver fibrosis (Wang et al., 2019b).

Several pathways inhibit TGF-β signaling, making them interesting as therapeutic strategies. Transcriptional intermediary factor 1γ, a negative regulator of the TGF-β pathway, interacts with Smad2/3 and binds to the promoter of the α-smooth muscle gene (α-SMA), downregulating α-SMA and activating HSCs (Lee et al., 2020). ECM1 interacts with αv integrins to keep TGF-β in an inactive form, thereby preventing HSC activation and liver fibrosis (Fan et al., 2019). Recent studies have explored epigenetic regulation of Smad- and non-Smad-mediated pathways in TGF-β signaling, highlighting the complex role of such signaling in fibrosis.

### Notch Signaling

Notch signaling is a conversed intercellular signaling pathway that regulates interactions between physically adjacent cells. Accumulating evidence suggests that Notch signaling participates in liver fibrosis by mediating myofibroblasts *trans*-differentiation and the EMT (Hu and Phan, 2016). When any one of five ligands (Delta-like1/3/4, Jagged-1/2) binds to the receptor for Notch1-4, the Notch intracellular domain (NICD) is released and translocates to the nucleus, where it binds to transcription factor CBF1/Suppressor of hairless/Lag1 (CSL) and modulates gene expression (Kovall and Blacklow, 2010). Notch activity in hepatocytes correlates with disease severity and treatment response in patients with NASH, and Notch is upregulated in a mouse model of diet-induced NASH and liver fibrosis. Forced activation of Notch in hepatocytes induces fibrosis by upregulating Sox9-dependent Osteopontin (Opn) secretion from hepatocytes, which activates resident HSCs (Zhu et al., 2018). Endothelial Notch1 overexpression results in LSEC dedifferentiation and accelerates liver fibrogenesis through eNOS-sGC signaling, and it alters the angiocrine profile of LSECs to compromise hepatocyte proliferation and liver regeneration (Duan et al., 2018). DLL4, a ligand of Notch signaling, is up-regulated in the LSECs of fibrotic liver of patients and of mice treated with CCl_4_, consistent with LSEC capillarization involving endothelin-1 (Chen et al., 2019a). Notch signaling is an attractive target for treating liver fibrosis; so far, Wnt/β-catenin signaling, miR-30c, liver fibrosis-associated lncRNA1 have been found to influence such signaling (Zhang et al., 2017; So et al., 2018; Gu et al., 2021). Notch signaling can also cross-talk with other signaling pathways involving TGF-β and Hedgehog, which together can regulate liver fibrosis (Xie et al., 2013; Wang et al., 2017; Fan et al., 2020).

Some compounds attenuate liver fibrosis by targeting Notch signaling and so may be novel potential therapeutic candidates for the treatment of liver fibrosis. For example, capsaicin shows liver fibrosis progression by regulating Notch signaling to reduce secretion of inflammatory cytokine TNF-α, which attenuates myofibroblast regeneration and fibrosis mediated by HSCs (Sheng et al., 2020). The natural sesquiterpene costunolide exerts potent antifibrotic effects by disrupting the WWP2/PPM1G complex, promoting Notch3 degradation and inhibiting the Notch3/HES1 pathway (Ge et al., 2020). The Notch inhibitor niclosamide exerts hepatoprotective effects against BDL-induced liver fibrosis (Esmail et al., 2021). Dibenzazepine, a bioavailable γ-secretase inhibitor and Notch antagonist, prevents activation of Notch receptors and is already in clinical trials as an anticancer treatment (Takebe et al., 2014). A nanoparticle system has been developed to deliver dibenzazepine to the liver for treatment of liver fibrosis and obesity-induced type 2 diabetes mellitus (Richter et al., 2020).

### Wnt Signaling

During liver fibrosis, canonical(β-catenin-dependent) and non-canonical (β-catenin-independent) pathways of Wnt signaling are activated and some proteins in the pathways are upregulated (Wang et al., 2018; Hu et al., 2020; Yu et al., 2020). In β-catenin-dependent pathways, Wnt ligation to cell surface receptors induces downstream phosphorylation and stabilization of β-catenin, which then translocates to the nucleus, where it acts together with p300 or CBP as a transcriptional co-activator of the T cell factor/lymphoid enhancer-binding factor (TCF/LEF) promoter (Miao et al., 2013; Lien and Fuchs, 2014; Nusse and Clevers, 2017). Non-canonical pathways comprise the β-catenin-independent planar cell polarity pathway and the non-canonical Wnt/Ca^2+^ pathways (De, 2011). Better understanding of Wnt signaling may provide novel insights into the pathophysiology of liver fibrosis.

Wnt also interacts with other pathways to influence liver fibrosis. For example, it blocks the phosphorylation of Smad3 and ERK to inhibit TGF-β1-induced *trans*-differentiation of fibroblasts into myofibroblasts (Liu et al., 2020a). The Wnt/β-catenin pathway may interact with the Smo-independent Gli1 pathway to promote HSC contraction via TCF4-dependent transrepression of Sufu (Zhang et al., 2021b). The “protein regulator of cytokinesis 1” (PRC1), which regulates the Wnt/β-catenin signaling pathway, may induce Gli1-dependent osteopontin expression to contribute to liver fibrosis (Rao et al., 2019). Wnt signaling may play a dual role in liver repair and liver ECM deposition: it promotes liver fibrosis in the BDL mouse model of liver fibrosis, but it protects the liver in the MDR2 KO mouse model of cholestatic liver disease (Jarman and Boulter, 2020). Thus, efforts to target the Wnt signaling to alleviate liver fibrosis should consider how to reduce scarring without affecting repair.

Numerous molecules have been identified that can inhibit Wnt signaling, such as antagonists, short interfering RNA (siRNA), soluble receptors, and the transcription inhibitors DKK1, ICG-001, PRI-724, and honokiol (Miao et al., 2013; Akcora et al., 2018; Nishikawa et al., 2018; Hu et al., 2020; Lee et al., 2021). These molecules may be candidate drugs for fibrosis treatment.

### YAP/TAZ Signaling

YAP/TAZ, a downstream effector of the alternative Wnt signaling pathway, is involved in liver fibrosis (Park et al., 2015). The YAP/TAZ-TEAD transcriptional complex plays an important role in the activity of the Hippo pathway (Bai et al., 2012; Crawford et al., 2018). YAP is activated in HSCs in patients with fibrotic livers, and inhibiting YAP using verteporfin impedes fibrogenesis in CCl_4_ mice (Mannaerts et al., 2015). Thus, inhibition of YAP may be a novel approach for treating fibrosis. Blockade of YAP reduces HSC activation and proliferation, while promoting their apoptosis. Loss of YAP also inhibits Wnt/β-catenin activity (Yu et al., 2019). Dynein-mediated interaction between YAP and acetylated microtubules may drive nuclear localization of YAP in the soft matrix, increasing TGF-β1-induced transcriptional activity of Smad for myofibroblast differentiation (You et al., 2020). Interestingly, activation of YAP attenuates hepatic damage and fibrosis in studies of liver ischemia-reperfusion injury, which may reflect the complex role of YAP in liver repair and fibrosis through processes such as Wnt signaling (Konishi et al., 2018; Liu et al., 2019b). The expression of YAP and TAZ in HSCs as well as hepatocytes promotes parenchymal inflammation and fibrosis (Mooring et al., 2020).

Verteporfin is the most commonly used small molecule inhibitor of YAP. Many other molecules alleviate fibrosis via inhibiting YAP/TAZ signaling, including magnesium isoglycyrrhizinate, acid ceramidease, dopamine receptor D1 agonist, and liquiritigenin (Li et al., 2018; Haak et al., 2019; Lee et al., 2019; Alsamman et al., 2020). Given its complex role in liver regeneration and HSC proliferation in different stages of NAFLD, balancing the activity of YAP in hepatocytes and HSCs during different disease stages is key for efficacy.

### Hedgehog Signaling

**FIGURE 4:**
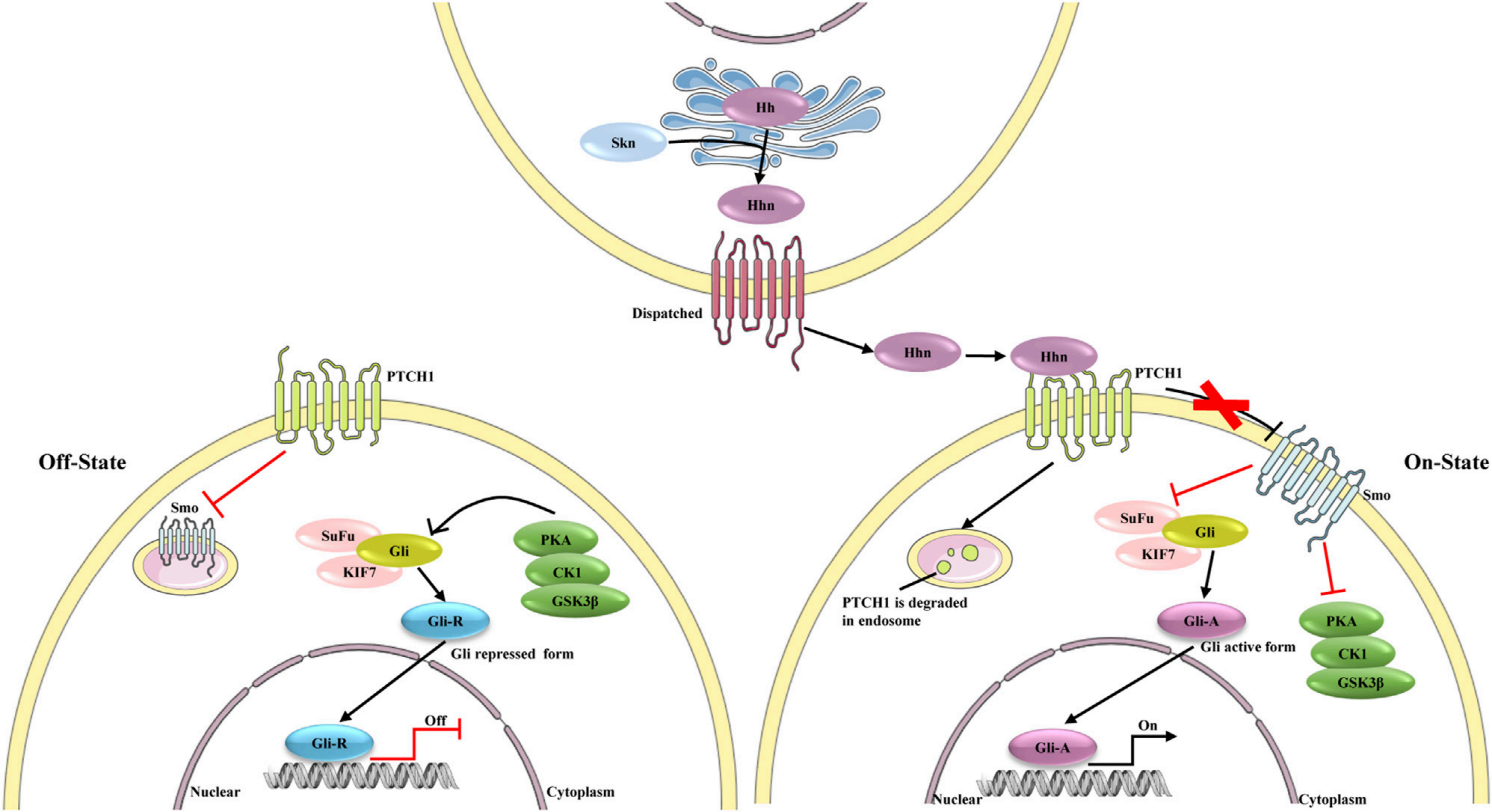
The different state of hedgehog signaling pathway. In the absence of ligands, the signaling is on “Off state”, PTCH1 inhibits the activity of Smo. Protein kinase (including PKA, CK1 and GSK3β), SuFu and KIF7 phosphorylate Gli, which leads proteolytic cleavage of Gli to Gli-repressor (Gli-R). Gli-R represses the expression of target genes. In Hhn secreting cells, the precursor of Hh is auto-cleaved and can be modified by a cholesterol at C-terminus to form Hhn on ER membrane. After this process, Hhn is secreted from the secreting cells and bind to PTCH1. PTCH1 is degraded in endosome, and consequently Smo repression is removed. Activated Smo inhibits the effect of PKA on Gli proteins, leading to the dissociation of SuFu, and Gli active form (Gli-A) is formed. Gli-A promotes the expression of target genes.

Growing evidence indicates that the hedgehog pathway is a critical regulator of adult liver repair and, hence, a potential diagnostic and/or therapeutic target in cirrhosis (Figure 4) (Machado and Diehl, 2018). Gli1 is the downstream transcriptional activator of hedgehog signaling, and it is also a marker of mesenchymal cells. Previous studies have confirmed perivascular Gli1^+^ mesenchymal-like cells to be a major driver of organ fibrosis (Kramann et al., 2015). Peribiliary Gli1^+^ mesenchymal cells are a subset of stromal cells characterized by active hedgehog signaling; these cells proliferate, acquire a myofibroblast phenotype, and surround the biliary tree in response to cholestatic injury, promoting liver fibrosis (Gupta et al., 2020). In fact, aberrant activation of hedgehog signaling in not only mesenchymal cells but also HSCs is considered crucial in liver fibrosis (Li et al., 2020a).

Some signaling factors and epigenetic modifications regulate hedgehog signaling and thereby influence HSC activation. These factors include lipopolysaccharide, palmitic acid, and the protein “predicted paired box 6” (Duan et al., 2017; Pan et al., 2017; Li et al., 2020a; Zhu et al., 2021), which may therefore be therapeutic targets in liver fibrosis. One study also suggested that miR-200a inhibits Gli3 expression and may function as a novel anti-fibrotic agent (Li et al., 2020b). Treating HSCs with the DNA methylation inhibitor 5-azadC prevents their proliferation and activation by restoring expression of Patched (PTCH1) (Yang et al., 2013). The metabolic state of HSCs affects their activation, and hedgehog signaling regulates metabolism (Chen et al., 2012; Du et al., 2018). Further work is needed to clarify exactly how hedgehog signaling regulates HSC activation and metabolism, thereby influencing liver.

Many chemical inhibitors of hedgehog inhibitors have been identified, including Gant61, GDC-0049, MD85, and vismodegib. These compounds have shown promise against liver fibrosis *in vivo* and *in vitro* (Li et al., 2019a; Kumar et al., 2019; Jiayuan et al., 2020). The naturally occurring iridoid glucoside geniposide, extracted from *Gardenia jasminoides* Ellis, inhibits hedgehog and thereby HSCs (Lin et al., 2019). These compounds are less effective against liver fibrosis in part because they cannot be delivered specifically to the liver. Two studies have achieved such delivery using nanoparticles, which improved drug efficacy. One group replaced the sulfonamide group of the hedgehog inhibitor GDC-0449 with two methylpyridine-2yl groups at the amide nitrogen, generating MDB5. This inhibitor was more potent at inhibiting hedgehog signaling and HSC proliferation *in vitro*. The research group also developed MDB5-loaded micelles, which enhanced systemic delivery of the drug and efficacy against liver fibrosis (Kumar et al., 2019). In another study, the hedgehog inhibitor vismodegib was loaded into cRGDyK-guided liposomes, which markedly inhibited the fibrogenic phenotype *in vivo*. The delivery system targeted the delivery of vismodegib to activated HSCs rather than quiescent HSCs, leading to preferential accumulation in fibrotic liver. These finding illustrate the promise of delivering therapeutic agents to activated HSCs to treat liver fibrosis (Li et al., 2019a).

### Fibroblast Growth Factor Signaling

Fibroblast growth factor (FGF) signaling is a prerequisite for adequate would healing, repair and homeostasis in various tissues and organs (Seitz and Hellerbrand, 2021). Since liver fibrosis is a wound healing response to liver injury, FGFs play an important role in hepatic fibrosis by acting as paracrine, and endocrine mediators of hepatocyte regeneration and HSC migration, proliferation and *trans*-differentiation (Schumacher and Guo, 2016). The paracrine FGFs (FGF1-10, FGF16-18, FGF20 and FGF22) bind strongly to heparan sulphate proteoglycans, which limits FGF diffusion through ECM and restricts their action to the site of secretion (Ornitz and Itoh, 2015; Seitz and Hellerbrand, 2021).

The three endocrine FGFs, FGF19 (mouse homolog FGF15), FGF21 and FGF23 participate in phosphate, bile acid, carbohydrate and lipid metabolism and thereby affect liver homeostasis (Itoh, 2010; Itoh et al., 2016; Kuro-o, 2019). Recent studies illustrate how FGF15/19 and FGF21 affect hepatic fibrosis and HSC activation. Hepatic accumulation of bile acid is central to the pathogenesis of cholestasis-induced liver injury, and excessive levels of cytotoxic bile acids in the liver can lead to liver fibrosis (Schaap et al., 2014). Expression of FGF15/19 is strongly induced by farnesoid X receptor (FXR) in the ileum, and the protein is secreted into the portal blood and transported to the liver, where it represses the expression of CYP7a1, a rate-limiting enzyme in bile acid synthesis, thereby mitigating liver fibrosis (Inagaki et al., 2005; Liu et al., 2020b). While enterocytes of the terminal ileum likely produce most FGF15/19, HSCs express FGFR4, while HSCs secrete FGF19. Enhanced FGF19/FGFR4 signaling blocks HSC proliferation and activation, which may help explain the anti-fibrotic effects of FGF19 observed in previous studies (Zhou et al., 2017; Hirschfield et al., 2019; Tian et al., 2021). FGF15 deficiency inhibits the development of hepatic fibrosis in animal models of NASH or liver fibrosis (Uriarte et al., 2015; Schumacher et al., 2017; Schumacher et al., 2020).

These findings suggest that FGF15/19 exert hepatoprotective effects via a pathway independent of bile acids. In contrast to FGF15/19, FGF21 is expressed predominantly in hepatocytes and is released in response to high levels of glucose and free fatty acids as well as low levels of amino acids. In this way, FGF 21 can prevent fatty liver, hepatic steatosis and hepatotoxicity (Reinehr et al., 2012; Seitz and Hellerbrand, 2021). FGF21 also inhibits HSC activation via TGF-β and NF-κB pathways, and it can induce HSC apoptosis through caspase-3, which attenuates hepatic fibrogenesis (Xu et al., 2016). FGF21 may participate in metabolism-related liver disease.

### Gas6 Signaling

Growth arrest-specific gene 6 (Gas6), a ligand of the TAM receptor (Tyro3, Axl, Mer), is a vitamin K-dependent protein expressed primarily by Kupffer cells, whereas Axl is found in both macrophages and quiescent HSCs in normal liver (Bárcena et al., 2015; Shrivastava et al., 2016). Serum levels of Gas6 correlate directly with liver stiffness and are significantly higher in patients with advanced fibrosis and primary biliary cholangitis (Bellan et al., 2016; Hayashi et al., 2020). In fact, the Gas6/TAM system has recently emerged as an important player in the progression of liver fibrosis and as a novel biomarker of liver fibrosis (Bellan et al., 2019; Smirne et al., 2019). CCl_4_-induced liver fibrosis activates the Gas6/Axl pathway, which in turn promotes HSC activation. Disrupting the pathway through Gas6 deficiency, Axl knockout or pharmacological inhibition attenuates hepatic fibrosis (Lafdil et al., 2006; Fourcot et al., 2011; Bárcena et al., 2015). Similarly, the rs4374383 polymorphism in the gene encoding Mer modulates HSC activation, affecting the severity of fibrosis in NAFLD (Petta et al., 2016). Gas6 also participates in both cardiac and pulmonary fibrosis by binding TAM (Espindola et al., 2018; Chen et al., 2019b; Li et al., 2019b). Targeting Gas6 signaling may be a potential treatment for liver fibrosis, so how Gas6 contributes to liver fibrosis should be further explored.

### Ferroptosis Signaling

Ferroptosis is a recently recognized form of regulated cell death, characterized by the presence of unusually small mitochondria with quite dense mitochondrial membrane, loss of mitochondria crista, rupture of the outer mitochondrial membrane, and accumulation of iron-based lipid reactive oxygen species (Xie et al., 2016). Ferroptosis is a defensive mechanism against cancer, neurotoxicity and ischemia/reperfusion-induced injury (Liang et al., 2019; Li et al., 2020c; Song and Long, 2020). In mice, an MCD diet induces iron accumulation, cell death and hepatic ferroptosis. Conversely, ferroptosis inhibitors alleviate MCD-diet induced inflammation, fibrogenesis and liver injury, suggesting an important role of ferroptosis in NASH (Li et al., 2020d). Ferroptosis is also an iron-dependent form of regulated cell death triggered by toxic lipid peroxidation, which is inhibited by glutathione peroxidase 4 (GPX4) in steatohepatitic liver (Qi et al., 2020).

Ferroptosis is now considered a new strategy for inhibiting HSCs to alleviate liver fibrosis (Zhang et al., 2018; Zhang et al., 2020a; Zhang et al., 2020b). How ferroptosis is regulated in HSCs remains unclear, although the RNA-binding protein ELAVL1/HuR, ZFP36/TTP, iron regulatory protein2 and the BRD7-p53-SLC25A28 complex appear to be involved. Ferroptosis can be trigged by inhibiting GPX4 (e.g., altretamine), inhibiting system Xc- (e.g., sorafenib, erastin, and sulfasalazine), depleting glutathione (e.g., BSO), or applying certain environmental conditions (e.g., high extracellular glutamate, amino acid starvation, cystine deprivation) (Alim et al., 2019). Thus, these treatments may be effective against liver fibrosis.

### cAMP-PKA-cAMP-Responsive Element-Binding Signaling

Cyclic cAMP (cAMP) is well-known as an antifibrogenic second messenger (Li et al., 2019c). The downstream cAMP-responsive element-binding (CREB) protein is a nuclear protein that binds to the cAMP-responsive element (CRE) in the promoter of the gene encoding neuropeptide, and CREB has been implicated in HSC activation and liver fibrosis (Cui et al., 2021). CREB-1, following its activation by phosphorylation, inhibits HSC proliferation and collagen expression *in vitro* (Deng et al., 2011), and it is involved in TGF-β3 auto-regulation in HSCs (Houglum et al., 1997). Phosphorylation or acetylation of CREB-1 in rat HSCs inhibits the TGF-β1 pathway, downregulating collagen I (Deng et al., 2016).

In contrast to these studies suggesting that CREB-1 can inhibit fibrosis, some studies indicate that it can promote hepatic fibrosis. One study, for example, suggested that phosphorylated CREB-1 promotes fibrosis by transactivating TGF-β1 expression (Wang et al., 2016b). Acetaldehyde can activate HSC-T6 cells, while caffeine can act via the adenosine A_2A_ receptor to inhibit the cAMP/PKA pathway and thereby suppress such activation (Wang et al., 2015). Blocking the interaction between CREB and β-catenin using the selective inhibitor PRI-724 reduces liver fibrosis induced by CCl_4_ or bile duct ligation (Osawa et al., 2015).

The potentially opposite effects of CREB in different cellular contexts suggest its complex involvement in liver fibrosis, which requires further investigation. Many studies have detected interaction between cAMP-PKA-CREB signaling and pathways mediated by TGF-β and Wnt. This may be a fruitful direction for future research. The molecules currently known to interact with CREB and to show therapeutic potential against liver fibrosis inhibit cAMP-PKA-CREB signaling. These molecules include ICG-001, a selective inhibitor of the CBP/β-catenin interaction (Henderson et al., 2010); PRI-724, which is phosphorylated on C-82 and is rapidly hydrolyzed *in vivo* into its active form, which shows acceptable toxicity and efficacy in preclinical studies (Lenz and Kahn, 2014; Osawa et al., 2015), and caffeine (Wang et al., 2015).

### Cellular Metabolism

Under normal circumstances, HSCs are in a resting state and show low metabolism. Their main function is to store small vitamin A fat droplets, which contain more than 70% of vitamin A in the body (Puche et al., 2013). Pro-inflammatory and pro-fibrotic cytokines can activate HSCs to release their stored vitamin A, proliferate, and produce ECM, with the cells adopting a high metabolic state (Chen et al., 2012; Higashi et al., 2017). In this way, HSCs undergo dramatic metabolic changes to meet the increased bioenergetic and biosynthetic demands of mitogenesis and ECM synthesis (Xie et al., 2015; Para et al., 2019; Zhao et al., 2020; Hewitson and Smith, 2021). These metabolic changes are often accompanied by increased glycolysis and mitochondrial respiration in order to optimize glucose consumption in HSCs and redirect them to support fibrogenic *trans*-differentiation (Lian et al., 2016).

Recent studies suggest that by changing the metabolism of activated HSCs, they can be converted into the resting type, offering opportunities for liver fibrosis treatment. In diseased livers of animals and patients, the number of glycolytic stromal cells is associated with the severity of fibrosis. Glycolysis is upregulated and lactate accumulates in quiescent HSCs that have been activated to become myofibroblasts. Hedgehog signaling regulates glycolysis to control the fate of HSCs (Chen et al., 2012). Increased aerobic glycolysis alone cannot meet the high metabolic demands of active HSCs: it works together with glutaminolysis (conversion of glutamine to α-ketoglutarate) to sustain energy metabolism and permit anabolism, and this is controlled by hedgehog signaling to YAP (Du et al., 2018). *In vivo*, glutaminolysis in HSCs is a marker of active fibrogenesis, and its cell-specific antagonism represents a potential therapeutic target by depriving the cells of glutamine (Du et al., 2020). Acetyl-CoA carboxylase (ACC), a regulator of fatty acid β-oxidation and *de novo* lipogenesis, has been implicated in metabolic reprogramming during HSC activation. ACC inhibitors prevent the *de novo* lipogenesis that is necessary for induction of glycolysis and oxidative phosphorylation during HSC activation, and such inhibitors thereby mitigate fibrosis (Bates et al., 2020). In addition to elevated levels of glutamine, elevated levels of fructose can increase risk of liver fibrosis (Song et al., 2019; Roeb and Weiskirchen, 2021). More studies are needed that explore metabolic regulation of HSCs, since this is a promising therapeutic strategy against liver fibrosis (Trivedi et al., 2021).

### Epigenetics

With the rise of advanced molecular methods, studies have begun to describe the epigenetic landscape of liver fibrosis, involving changes in DNA methylation, histone modifications and levels of non-coding RNAs that control chromatin structure and DNA accessibility to the transcriptional machinery. DNA methylation is carried out by three enzyme: DNA methyltransferase 1 (DNMT1), DNMT3A and DNMT3B (Jin et al., 2011). MCP2 influences methylation of the gene encoding PPARγ, leading to its silencing, which in turn promotes HSC activation (Mann et al., 2010). DNMT1 and DNMT3B methylates the genes encoding regulator of calcineurin 1 (RCAN1), prostacyclin synthase (PTGIS), Septin9 and SAD1/UNC84 domain protein-2 (SUN2), promoting HSC activation and liver fibrosis (Wu et al., 2017; Chen et al., 2018; Pan et al., 2018; Pan et al., 2019). These findings suggest that gene methylation is important for HSC activation. In fact, DNA methylation affects other epigenetic process, including the expression and activity of long non-coding RNAs, which in turn influence HSC activation and fibrosis. One example is the DNMT1-LncRNA H19 epigenetic pathway, which is involved in HSC activation and liver fibrosis (Yang et al., 2018a). Hypermethylation of the gene encoding PSTPIP2 not only activates HSCs but also polarizes macrophages in mice with CCl_4_-induced hepatic fibrosis (Yang et al., 2018b).

It is not surprising, then, that the various histone modifications, which include methylation, acetylation, phosphorylation, ubiquitination, deamination, and sumoylation, are considered targets for fibrosis treatment (El Taghdouini and van Grunsven, 2016). For example, the enhancer of zeste homologue 2 (EZH2), which is responsible for the trimethylation of histone 3 at lysine 27(H3K27me3), is involved in TGF-β dependent fibrogenic pathways (Martin-Mateos et al., 2019). EZH2 and the demethylase JMJD3 regulate HSC activation and liver fibrosis (Jiang et al., 2021). Histone deacetylases 1/2 (HDAC1/2) may regulate liver fibrosis and may therefore be therapeutic targets (Liu et al., 2021; Zhu et al., 2021). Many genes are regulated through cross-talk between histone and DNA mehyltransferases such as G9a and DNMT1. CM272, a first-in-class reversible inhibitor of G9a and DNMT1, can halt fibrogenesis without causing toxic effects (Barcena-Varela et al., 2021).

Numerous non-coding RNAs such as microRNAs and long non-coding RNAs play important roles in liver fibrosis. For example, miR-199a, miR-200a/b, miR-122, miR-194/192, miR-223, miR-21, miR-155 and miR-29 are expressed or enriched in several types of hepatic cells or in the circulation specifically in the presence of liver disease, implying that they play important roles in pathogenesis (Murakami et al., 2011; Wang et al., 2021). Nanoparticle-based delivery of miR-30c to LSECs inhibits the DLL4/Notch pathway and angiogenesis, ameliorating liver fibrosis *in vivo* (Gu et al., 2021). The lncRNA-ATB is upregulated in fibrotic liver tissues and activated LX‐2 cells. Knockdown of lncRNA‐ATB downregulates β‐catenin by upregulating the endogenous miR‐200a and suppressing activation of LX‐2 cells (Fu et al., 2017). HOTAIR act as an endogenous “sponge” for miR‐148b to facilitate expression of DNMT1, which in turn promotes HSC proliferation and activation (Bian et al., 2017).

Several inhibitors of DNA methylation (e.g., 5-azadC, Sennoside A) and histone modifications (e.g., givinostat, DZNep, GSK-503, GSK-J4) as well as epigenetic inhibitors such as CM272 have shown promise for treating liver fibrosis (Yang et al., 2013; Martin-Mateos et al., 2019; Zhu et al., 2020; Barcena-Varela et al., 2021; Ding et al., 2021; Huang et al., 2021; Jiang et al., 2021). Epigenetic biomarkers may be useful not only as treatment targets but also for assaying in tissue and liquid biopsies in order to predict prognosis of patients with liver fibrosis. For example, the levels of H3K27ac in specific oncogenes and of TS, PPARγ-mediated DNA methylation have been suggested for this purpose (Hardy et al., 2017; Arechederra et al., 2021; Jühling et al., 2021).

## Candidate Drugs During Clinical Trails for Liver Fibrosis

Recently, with the in-depth understanding of the pathogenesis of liver fibrosis, some new compounds with anti-fibrosis potential have emerged and are in clinical trial (Rotman and Sanyal, 2017; Lambrecht et al., 2020; Attia et al., 2021) (Table 1). The therapeutic targets of these compounds contain metabolism, gut-liver axis, inflammation and cell death, which share their effects among whole body. After fully confirming drug’s efficacy on liver fibrosis, finding a suitable targeted delivery system for drugs may help for its clinical use with better treatment efficacy and lower side effects. The future candidate drugs for liver fibrosis may develop from the novel targets or its combinatorial use.

**TABLE 1:** New pharmacotherapeutics with antifibrotic effects currently in clinical trial.

Drugs	Mechanism	Therapeutic target	Current status
Saroglitazar	PPARα/γ agonist	Metabolism	Phase 2
K-877	PPARα agonist	Metabolism	Phase 2
Elafibranor	PPAR α/δ agonist	Metabolism	Phase 2
IVA337	PPARα/δ/γ agonist	Metabolism	Phase 2
BMS-986036	FGF21 analogue	Metabolism	Phase 2
LIK066	SGLT1/2 inhibitor	Metabolism	Phase 2
GS-0976	ACC1/2 inhibitor	Metabolism	Phase 2
Liraglutide	GLP-1 receptor agonist	Gut-liver	Phase 2
Cotadutide	GLP1/glucagon receptor agonist	Gut-liver	Phase 2
Semaglutide	GLP-1 receptor agonist	Gut-liver	Phase 2
Aldafermin (NGM282)	FGF19 analogue	Gut-liver	Phase 2
Balapectin	Galectin-3 inhibitor	Inflammation	Phase 2
Selonsertib (GS-4997)	ASK1 inhibitor	Cell death	Phase 3
Emricasan	Caspase inhibitor	Cell death	Phase 3
MGL-3196	THR-β agonists	Metabolism	Phase 3
Obeticholic Acid	FXR agonist	Metabolism	Phase 3
Aramchol	Scd-1 inhibitor	Metabolism	Phase 3
Cenicriviroc	CCR2/5 antagonist	Inflammation	Phase 3

## Conclusion

In this review, we have outlined how major types of hepatic cells participate in liver fibrosis, and we have described several novel targets for fibrosis therapy. Our hope is to provide directions for future investigations.

Early research on the mechanism of fibrosis has focused mainly on HSC activation and collagen deposition. More recent research has focused on cellular state and processes, including metabolism, HSC proliferation and apoptosis, and epigenetic modifications. These studies have broadened our understanding of the pathogenesis of fibrosis, and have pointed out new directions for research into anti-fibrotic drugs. The many in-depth studies on the pathogenesis of liver fibrosis have identified novel signaling pathways as well as signaling crosstalk between TGF-β and Wnt/β-catenin, TGF-β and hedgehog, or YAP and hedgehog.

These studies will gradually build a complete picture of the pathogenesis of fibrosis and provide new ideas in the search for treatment targets. These studies highlight that exploiting crosstalk between signaling pathways may lead to the development of more effective drugs against liver fibrosis.
